# Supplementary material for: End Sequence Analysis Toolkit (ESAT) expands the extractable information from single-cell RNA-seq data
Source: Genome Res. 2016 Oct;26(10):1397–410. doi: 10.1101/gr.207902.116 (PMC5052061; doi:10.1101/gr.207902.116)
Supplement: Supplemental Material [file supp_gr.207902.116_Supp_Materials.tar.gz › ESAT-esat_v0.1/doc/html/classbroad_1_1core_1_1multiplealignment_1_1_phylip_interleaved_multiple_alignment_i_o.html]

ESAT: broad.core.multiplealignment.PhylipInterleavedMultipleAlignmentIO Class Reference


ESAT


- Main Page
- Related Pages
- Packages
- Classes
- Files

- Class List
- Class Index
- Class Hierarchy
- Class Members

All Classes Namespaces Files Functions Variables Enumerator Pages

- broad
- core
- multiplealignment
- PhylipInterleavedMultipleAlignmentIO

Public Member Functions |
List of all members

broad.core.multiplealignment.PhylipInterleavedMultipleAlignmentIO Class Reference

Inheritance diagram for broad.core.multiplealignment.PhylipInterleavedMultipleAlignmentIO:

[legend]

Collaboration diagram for broad.core.multiplealignment.PhylipInterleavedMultipleAlignmentIO:

[legend]

|  |  |
| --- | --- |
| Public Member Functions | |
| MultipleAlignment | load (String source) throws IOException, ParseException |
|  | |
| MultipleAlignment | load (String fileName, List< String > sequencesToLoad) throws IOException, ParseException |
|  | |
| MultipleAlignment | load (InputStream in) throws IOException, ParseException |
|  | |
| MultipleAlignment | load (InputStream in, List< String > sequencesToLoad) throws IOException |
|  | |
| void | setSpacing (int spacing) |
|  | |
| void | write (BufferedWriter bw, MultipleAlignment ma) throws IOException |
|  | |
| void | write (BufferedWriter bw, MultipleAlignment ma, List< String > order) throws IOException |
|  | |
| String | getPreferredFileExtension () |
|  | |

## Member Function Documentation

|  |  |  |  |  |
| --- | --- | --- | --- | --- |
| String broad.core.multiplealignment.PhylipInterleavedMultipleAlignmentIO.getPreferredFileExtension | ( |  | ) |  |

Implements broad.core.multiplealignment.MultipleAlignmentIO.

|  |  |  |  |  |  |
| --- | --- | --- | --- | --- | --- |
| MultipleAlignment broad.core.multiplealignment.PhylipInterleavedMultipleAlignmentIO.load | ( | String | *source* | ) | throws IOException, ParseException |

Implements broad.core.multiplealignment.MultipleAlignmentIO.

Here is the caller graph for this function:

|  |  |  |  |
| --- | --- | --- | --- |
| MultipleAlignment broad.core.multiplealignment.PhylipInterleavedMultipleAlignmentIO.load | ( | String | *fileName*, |
|  |  | List< String > | *sequencesToLoad* |
|  | ) |  | throws IOException, ParseException |

Implements broad.core.multiplealignment.MultipleAlignmentIO.

Here is the call graph for this function:

|  |  |  |  |  |  |
| --- | --- | --- | --- | --- | --- |
| MultipleAlignment broad.core.multiplealignment.PhylipInterleavedMultipleAlignmentIO.load | ( | InputStream | *in* | ) | throws IOException, ParseException |

Implements broad.core.multiplealignment.MultipleAlignmentIO.

Here is the call graph for this function:

|  |  |  |  |
| --- | --- | --- | --- |
| MultipleAlignment broad.core.multiplealignment.PhylipInterleavedMultipleAlignmentIO.load | ( | InputStream | *in*, |
|  |  | List< String > | *sequencesToLoad* |
|  | ) |  | throws IOException |

Implements broad.core.multiplealignment.MultipleAlignmentIO.

|  |  |  |  |  |  |
| --- | --- | --- | --- | --- | --- |
| void broad.core.multiplealignment.PhylipInterleavedMultipleAlignmentIO.setSpacing | ( | int | *spacing* | ) |  |

|  |  |  |  |
| --- | --- | --- | --- |
| void broad.core.multiplealignment.PhylipInterleavedMultipleAlignmentIO.write | ( | BufferedWriter | *bw*, |
|  |  | MultipleAlignment | *ma* |
|  | ) |  | throws IOException |

Implements broad.core.multiplealignment.MultipleAlignmentIO.

Here is the caller graph for this function:

|  |  |  |  |
| --- | --- | --- | --- |
| void broad.core.multiplealignment.PhylipInterleavedMultipleAlignmentIO.write | ( | BufferedWriter | *bw*, |
|  |  | MultipleAlignment | *ma*, |
|  |  | List< String > | *order* |
|  | ) |  | throws IOException |

Implements broad.core.multiplealignment.MultipleAlignmentIO.

Here is the call graph for this function:

---

The documentation for this class was generated from the following file:

- java/broad/core/multiplealignment/PhylipInterleavedMultipleAlignmentIO.java


---

Generated on Fri Aug 15 2014 15:10:55 for ESAT by  

 1.8.7
